# Linking public health agencies and hospitals for improved emergency preparedness: North Carolina's public health epidemiologist program

**DOI:** 10.1186/1471-2458-12-141

**Published:** 2012-02-23

**Authors:** Milissa Markiewicz, Christine A Bevc, Jennifer Hegle, Jennifer A Horney, Megan Davies, Pia DM MacDonald

**Affiliations:** 1 North Carolina Preparedness and Emergency Response Research Center, North Carolina Institute for Public Health, Gillings School of Global Public Health, University of North Carolina, Chapel Hill, North Carolina, USA; 2 Department of Epidemiology, Gillings School of Global Public Health, University of North Carolina, Chapel Hill, North Carolina, USA; 3 Epidemiology Section, North Carolina Division of Public Health, North Carolina Department of Health and Human Services, Raleigh, North Carolina, USA

## Abstract

**Background:**

In 2003, 11 public health epidemiologists were placed in North Carolina's largest hospitals to enhance communication between public health agencies and healthcare systems for improved emergency preparedness. We describe the specific services public health epidemiologists provide to local health departments, the North Carolina Division of Public Health, and the hospitals in which they are based, and assess the value of these services to stakeholders.

**Methods:**

We surveyed and/or interviewed public health epidemiologists, communicable disease nurses based at local health departments, North Carolina Division of Public Health staff, and public health epidemiologists' hospital supervisors to 1) elicit the services provided by public health epidemiologists in daily practice and during emergencies and 2) examine the value of these services. Interviews were transcribed and imported into ATLAS.ti for coding and analysis. Descriptive analyses were performed on quantitative survey data.

**Results:**

Public health epidemiologists conduct syndromic surveillance of community-acquired infections and potential bioterrorism events, assist local health departments and the North Carolina Division of Public Health with public health investigations, educate clinicians on diseases of public health importance, and enhance communication between hospitals and public health agencies. Stakeholders place on a high value on the unique services provided by public health epidemiologists.

**Conclusions:**

Public health epidemiologists effectively link public health agencies and hospitals to enhance syndromic surveillance, communicable disease management, and public health emergency preparedness and response. This comprehensive description of the program and its value to stakeholders, both in routine daily practice and in responding to a major public health emergency, can inform other states that may wish to establish a similar program as part of their larger public health emergency preparedness and response system.

## Background

Both hospitals and public health agencies play a critical role in detecting and responding to public health emergencies. Since 2001, collaboration between hospitals and public health agencies for improved emergency preparedness has been encouraged by cooperative grants issued by the U.S. Centers for Disease Control and Prevention (CDC) and the Health Resources and Services Administration [1]. In 2005 and 2006, the American Public Health Association and American Medical Association convened joint leadership summits focused on the lack of linkages between acute care and the public health system with regard to preparedness. These summits resulted in a call for action, including a specific recommendation to "establish effective, real-time data systems to capture and share medical and public health information. This includes... improvement of syndromic surveillance systems, disease and injury reporting, and... connectivity of public health and EMS agencies and hospitals." [2]

North Carolina's direct involvement in the 2001 anthrax investigations, and the state's previous experience assessing medical consequences resulting from Hurricane Floyd in 1999, required close collaboration between the North Carolina Division of Public Health (NCDPH), local health departments (LHDs), and many of the state's hospitals. In both events, state and local public health officials worked closely with hospital staff to conduct syndrome-based surveillance in response to a public health emergency. Several factors hindered the efforts of investigators. These included the limited relationships among organizations (hospitals and public health agencies) which resulted in less than ideal familiarity with each other's capacity, inefficient communication and information sharing, and, in the case of anthrax, the need to educate healthcare providers about the epidemiology of inhalational anthrax [3].

Recognizing the need for more efficient communication and collaboration between hospitals and public health agencies to better respond to future public health emergencies, NCDPH established an innovative program in 2003 that placed public health epidemiologists (PHEs) in hospitals around the state. Funded by the state's Public Health Emergency Preparedness cooperative agreement with CDC, PHEs are employees of their respective hospitals, serving NCDPH and LHDs as preferential partners.

As of 2010, eleven PHEs were based in the state's largest hospitals (including a Veterans Affairs Medical Center), covering approximately 39% of general/acute care beds and 30% of emergency department visits. The PHE program serves to "develop a communications infrastructure to facilitate and ensure the timely dissemination and transfer of information between the healthcare and public health sectors" as described in the HHS Pandemic Influenza Plan [4]. PHEs are responsible for: 1) surveillance, detection, and monitoring of community-acquired infections and potential bioterrorism events; 2) assisting LHDs with public health investigations; 3) educating clinicians regarding diseases of public health importance; 4) enhancing communication among clinicians, hospitals, and the public health system; and, 5) conducting special studies. In April 2010, the PHE program was recognized as a "promising practice" by the Center for Infectious Disease Research and Policy for aiding North Carolina's H1N1 response by shortening emergency response time [5].

The PHE program is a unique collaborative effort between hospitals and public health agencies that may be of interest to other states seeking to create similar linkages. Researchers at the North Carolina Preparedness and Emergency Response Research Center (NCPERRC) undertook a study of the PHE program in 2010 to 1) identify the specific activities carried out by PHEs and the services they provide to three stakeholder groups-LHDs, NCDPH, and the hospitals in which they are based, 2) determine the value of these services to stakeholders, and 3) describe PHEs' role in North Carolina's response to the 2009 novel influenza A (H1N1) pandemic.

## Methods

To document PHEs' activities and the services they provide to stakeholders in routine daily practice and during the H1N1 pandemic, data were collected from PHEs and staff based at LHDs, NCDPH, and hospitals. PHEs completed an electronic (SurveyMonkey^®^) survey and individual semi-structured interviews. Survey items and interview questions focused on eliciting the specific activities associated with each of the PHE's 5 broad areas of responsibility including surveillance, assisting LHDs, educating clinicians, enhancing communication, and conducting special studies.

As PHEs' contact with LHDs occurs primarily through communicable disease and tuberculosis (TB) control nurses, the lead individuals in these positions were surveyed regarding the importance of services provided by PHEs to LHDs, length of time for PHEs to respond to LHD requests, and impact of the PHE program on LHDs' capacity to detect and investigate communicable disease outbreaks and potential bioterrorism events. A link to an electronic (SurveyMonkey^®^) survey was sent to 150 lead communicable disease and TB control nurses based in the state's 85 LHDs; 6 surveys were returned due to non-deliverable email addresses. The final sample size (N) for this survey was 144.

Semi-structured interviews were conducted with key informants at NCDPH who work closely with PHEs. Interviews focused on the specific ways NCDPH uses the network of PHEs for ongoing state-based syndromic surveillance and communicable disease management, the perceived value of the PHE program, and how NCDPH utilized the PHEs to respond to the H1N1 pandemic. Semi-structured interviews were also conducted with PHEs' hospital supervisors. Interviews focused on the services PHEs provide to their host hospitals and how valuable these services are to the hospital's ability to prepare for and respond to public health emergencies, such as the H1N1 pandemic.

All interviews were recorded, transcribed, and imported into ATLAS.ti for coding and analysis. Quantitative interview data, and data from completed surveys, were entered into Microsoft Excel for descriptive analysis. The study was found exempt from review by the University of North Carolina Institutional Review Board (09-0523).

## Results

Ten PHEs completed the electronic survey and individual interview; the eleventh position was vacant at the time of the study (response rate = 100%). The 10 PHEs surveyed had been in their positions for an average of 49 months at the time of the study (range, 17-80 months). All PHEs held a minimum of a BA or BS degree; fields of study included biology (3), nursing (3), medical technology (2), sociology (1), and environmental health science (1). Eight PHEs held one or more master's degrees; fields of study included public health (3), microbiology (2), education (2), health care administration (1), and nursing (1). One PHE held a PhD in epidemiology. PHEs also reported training in infection control, communicable disease, incident command systems, forensic epidemiology, and risk communication. Nine PHEs were based in their hospital's infection control department, while the tenth was based in their medical center's infectious diseases section.

Of the 144 communicable disease and TB control nurses surveyed, 119 completed the electronic survey (response rate = 82.6%). These 119 nurses represented 74 (87.1%) of the state's 85 LHDs. Respondents indicated they had been in their current positions for an average of 7 years (range, 2 months-42 years). Of the 119 nurses that completed the survey, 88 (73.9%) had interacted with one or more PHEs on a professional basis in the past year (August 2009-July 2010).

Four NCDPH key informants were interviewed (2 program managers and 2 epidemiologists). All 11 hospital supervisors were interviewed (response rate = 100%). Hospital supervisors held positions as the director, assistant director, or manager of infection control (8), infectious disease physician (1), hospital epidemiologist (1), and chief medical officer (1).

### Services provided by public health epidemiologists

PHEs estimated the time spent on each of their 5 areas of responsibility and listed the activities associated with each. Surveys and/or interviews with LHD-based nurses, NCDPH key informants, and PHEs' hospital supervisors confirmed these activities.

PHEs spend the largest percentage of their time (mean, 46%; range, 30%-50%) on activities related to surveillance. PHEs use North Carolina's statewide syndromic surveillance system-the North Carolina Disease Event Tracking and Epidemiologic Collection Tool (NC DETECT)-to monitor emergency department visits for their hospital system and investigate "signals" (i.e., increases in syndromes above pre-established thresholds [6]). All PHEs have received training on the use of NCDETECT. In addition, PHEs actively monitor their hospital's admissions, lab, and death reports on a daily basis. When a case/cluster of interest is detected, PHEs access patient medical records to investigate if it represents a real event.

NCDPH utilizes the network of PHEs to support specific state surveillance objectives. At the time of the study, PHEs were conducting febrile respiratory illness admission surveillance and laboratory surveillance for viral respiratory pathogens. In addition, PHEs prioritize surveillance for specific diseases based on information received from NCDPH or LHDs about cases/clusters of communicable disease in the community or state.

PHEs spend the second largest percentage of their time assisting LHDs with communicable disease reporting and investigation (mean, 20%; range 10%-40%). PHEs report communicable disease cases to LHDs either directly or by assisting physicians, respond to LHD requests (e.g., for information from patients' medical records), facilitate access to physicians, alert LHDs of unusual cases/clusters among inpatients or emergency department outpatients, and perform descriptive epidemiology on clusters.

In addition, PHEs send weekly reports to LHDs on communicable disease cases at their hospital and disseminate similar weekly influenza reports during flu season. PHEs may also meet with nearby LHDs' "epidemiology teams" (multidisciplinary public health response teams) to review cases of epidemiological significance.

An average of 13% of PHEs' time is spent enhancing communication (range, 5%-30%). In most instances, activities listed under "assisting LHDs" and "educating clinicians" serve the dual purpose of enhancing communication. Moreover, PHEs enhance communication by providing NCDPH with nearly all of the same services they provide to LHDs (i.e., reporting cases and assisting with investigations). PHEs also provide NCDPH with a channel for disseminating information (e.g., CDC advisories, state guidance) to hospitals. PHEs further enhance communication by serving on hospital disaster preparedness committees, representing public health views and concerns.

PHEs spend an average of 9% of their time educating clinicians (range, 5%-20%) by providing annual or as needed training to medical students, residents, and clinicians on communicable disease reporting and infection control. PHEs also deliver in-service presentations to hospital clinicians regarding diseases of public health significance.

Conducting special studies accounts for an average of 9% of PHEs' time (range, 2%-15%). Examples of special studies include a PHE-led study of diagnosis codes for surveillance, participation in CDC/national studies, and hospital-initiated studies (e.g., outbreak of norovirus infection among bone transplant patients).

### Value of the public health epidemiologist program

Interviews and surveys provided an opportunity for respondents from LHDs, NCDPH, and hospitals to rate and/or discuss the perceived value of the PHE program. LHD-based nurses who responded to the electronic survey rated the importance of 10 services received from PHEs (see Table 1). All ten services were rated as very or somewhat important by over 87% of respondents. Respondents reported that information requested for public health investigations was received more quickly from PHEs than from staff at "non-PHE" hospitals (i.e., all other hospitals in the state except the 11 that house PHEs). Nearly 40% (39.8%) of respondents reported receiving requested information immediately from PHEs, while only 15.7% received requested information immediately from infection control practitioners (ICPs) or medical records staff at non-PHE hospitals.

**Table 1:** Importance of Services Provided by Public Health Epidemiologists to Local Health Departments

Service	Very important (%)	Somewhat important (%)	Not important (%)
Respond directly to LHD's requests for information needed from a patient's medical record for reporting or investigation purposes.	100.0	0.0	0.0
Report cases of CD at their hospital to LHD for patients that reside in county or health district.	99.2	0.8	0.0
Proactively inform LHD of unusual cases/clusters of CD at their hospital.	94.1	5.9	0.0
Facilitate LHD's access to physicians or others at their hospital who can provide information needed from a patient's medical record for reporting or investigation purposes.	94.1	5.9	0.0
Refer patients (or family members of patients) with a CD for follow-up services, as needed.	91.5	7.6	0.8
Pass on new or timely information from NCDPH, their hospital, and/or CDC regarding diseases of public health importance.	72.9	25.4	1.7
Conduct interviews with patients and/or their family members at LHD's request.	63.6	30.5	5.9
Provide regular reports on influenza cases at their hospital during flu season.	56.8	38.1	5.1
Meet regularly with LHD staff to review reportable cases, provide updates, and/or share information.	42.4	49.2	8.5
Meet with LHD's Epidemiology Team to review cases, provide updates, and/or share information.	36.4	50.8	12.7

Abbreviations: *LHD *local health department; *CD *communicable disease; *NCDPH *North Carolina Division of Public Health; *CDC *Centers for Disease Control and Prevention.

To further gauge LHDs' perception of the value of the PHE program, nurses rated the impact of the PHE program on four measures. Of the respondents that interacted with a PHE in the past year, 85% or more reported that the PHE program either greatly or somewhat enhanced 1) communication between hospitals and LHDs, 2) the timeliness of communicable disease reporting, 3) the completeness of communicable disease reporting, and 4) their LHD's efficiency in reporting and investigating communicable disease in the community.

NCDPH key informants placed a high value on the PHE program's ability to enhance the timeliness of case reporting and response, sensitivity/specificity of syndromic surveillance, and communication with hospitals. One key informant reported that the PHE program has increased the flow of information around communicable disease reporting and investigation, allowing for timelier interventions. PHEs immediately report significant cases to NCDPH, and can quickly access and share needed information from patient medical records, thereby increasing the speed of response. A second key informant highlighted the value of PHE program in enhancing surveillance, noting that surveillance information gathered by PHEs has been more specific than data available through the NC syndromic surveillance system. "Having a human being looking at cases and excluding the ones that have been clearly attributed to a non-flu cause is helpful and that is something we cannot do with [the syndromic surveillance system]." Key informants also emphasized the value of PHEs in connecting NCDPH to hospitals. PHEs provide "on the ground access to the issue of the day... and [act as] our liaison for dealing with the situation."

In discussing the value of the program to hospitals, PHEs' supervisors highlighted two unique aspects of the PHE role-its focus on syndromic surveillance of community-acquired infections and bioterrorism events, and its connection to public health agencies. Supervisors noted that this role is distinct from that of ICPs, who focus on hospital-acquired infections.

Eight supervisors noted that PHEs had played a significant role in responding to a public health emergency or outbreak (other than H1N1), including an *E. coli *outbreak at the state fair, illness related to contaminated street drugs, and various localized outbreaks of norovirus infection, pertussis, shigellosis, and cryptosporidiosis. Supervisors reported that PHEs often identified the outbreak and took an active role in responding by sharing key information (e.g., case definition, exposures) with frontline responders (e.g., emergency department and clinic staff). Using a scale of 1 to 10, supervisors rated the role played by their PHE in responding to public health emergencies or outbreaks (other than H1N1) as highly valuable (mean, 9.6; range, 8-10).

Supervisors also emphasized the value of the PHE position in providing a bridge between their hospital and public health agencies. Supervisors noted that PHEs' connection to public health resources allows hospitals to stay abreast of national trends, guidelines, and best practices regarding the management of diseases of public health significance. In addition, PHEs help "hospital staff to have a better comprehension of the role of public health and how we need to partner to promote the wellness and health of the community as a whole." Sub- heading for this section

### Role of public health epidemiologists in the 2009 H1N1 pandemic response

The specific activities carried out by PHEs during the H1N1 pandemic are listed in Table 2. Approximately half of all LHD-based nurses surveyed interacted with a PHE. Over 85% of these respondents reported that that PHEs either greatly or somewhat enhanced communication between hospitals and LHDs with regard to H1N1 reporting and investigation, the timeliness and completeness of H1N1 reporting, and their LHD's efficiency in reporting and investigating H1N1 in the community (see Table 3).

**Table 2:** Public Health Epidemiologists' Role in the 2009 H1N1 Pandemic Response

Responsibility	Activities
Surveillance, detection, and monitoring of H1N1	• Heightened surveillance of influenza-like illnesses • Kept a running list of possible and confirmed H1N1 cases • Monitored possible and confirmed H1N1 cases for outcomes • Received test results from state lab and informed physicians of results

Assisting LHDs	• Reported cases • Provided daily/weekly reports on the status of H1N1 • Assisted LHDs with obtaining contact information when LHDs were heavily engaged in contact tracing • Worked closely with LHDs to facilitate transportation of samples to the state public health lab

Educating clinicians on H1N1	• Educated physicians and other hospital staff on symptoms of H1N1, how to isolate patients with possible H1N1, the type of respiratory protection to use, and how to collect swabs from possible cases • Educated hospital staff on importance of receiving the H1N1 vaccine • Provided hospital staff with daily/weekly H1N1 status reports • Served on H1N1 task forces and committees • Provided data and information needed to develop hospital policies and educated staff on these policies

Enhancing communication among hospitals and public health	• Participated in NCDPH-led conference calls on H1N1 • Provided NCDPH with daily/weekly H1N1 status reports

Special studies	• Participated in CDC, NCDPH, and/or hospital-initiated studies on H1N1 deaths, H1N1 and pregnant or postpartum women, and H1N1 with seizure complications

Abbreviations: *LHD(s) *local health department(s); *NCDPH *North Carolina Division of Public Health; *CDC *Centers for Disease Control and Prevention.

**Table 3:** Impact of Public Health Epidemiologists on Local Health Department Reporting and Investigation of H1N1

Measure	Greatly enhanced (%)	Somewhat enhanced (%)	Did Not enhance (%)	Response count (No.)
Communication between hospitals and local public health with regard to H1N1 reporting and investigation	70.7	17.2	12.1	58
Completeness of H1N1 reporting	58.9	26.8	14.3	56
Timeliness of H1N1 reporting	66.1	21.4	12.5	56
LHD's ability to be more efficient in reporting and investigating cases/clusters of H1N1	62.5	23.2	14.3	56

Abbreviation: *LHD *local health department

In discussing the value of PHEs in responding to H1N1, NCDPH key informants emphasized PHEs' ability to provide timely surveillance data to NCDPH and facilitate communication with hospitals. One NCDPH key informant noted, "They were really our link to those hospitals...[without the PHEs] we wouldn't have had a good sense of how many cases they're seeing, how many cases are in ICU [intensive care unit], how many cases among pregnant women and kids... it guided our recommendations on surveillance, testing, and infection control." A second key informant reported that PHEs "were integral in communicating case definitions and guidance information on treatment, isolation, and quarantine to their hospitals." NCDPH informants also valued PHEs' flexibility in quickly adapting the focus of their surveillance efforts to meet changing needs and priorities during the pandemic. "It didn't take time at all for them if requirements were changed. For example, monitor acute respiratory admissions, then monitor death, then monitor pregnant women with influenza, or monitor ICU admissions."

PHEs' hospital supervisors placed a high value on the role played by their PHE in responding to the pandemic, giving them an average score of 9.1 (range, 5-10) on a scale of 1 to 10. Hospital supervisors reported that PHEs were instrumental in helping develop hospital policies/protocols for the management of H1N1 based on surveillance data and state and federal guidance, and educating hospital staff on these policies/protocols. Having a PHE "allowed us to be linked with other PHEs and other resources in the state...[and] with the federal folks so that we could ensure that we were aware of what was going on and our response to what was going on in the region was the most appropriate thing to do at the time."

## Discussion

Several reports and studies have highlighted the lack of critical linkages between the healthcare and public health systems for emergency preparedness and response [2,3,7,8]. The findings of this study suggest that in North Carolina, PHEs have effectively linked public health agencies and hospitals for enhanced syndromic surveillance and communicable disease management. In doing so, PHEs have improved emergency response capability in North Carolina, as demonstrated by their role in the 2009 H1N1 pandemic response.

For syndromic surveillance systems to be effective in detecting outbreaks and potential bioterrorism events in their earliest stages, signals must be investigated in real-time [9]. Because PHEs are hospital employees focused on diseases of public health significance, they are uniquely positioned to quickly and accurately investigate signals from North Carolina's syndromic surveillance system for their hospitals, which account for 30% of emergency department visits in North Carolina. To confirm or rule out potential cases, PHEs have the ability to immediately access confidential patient medical records and can also consult with emergency department and infectious disease physicians, infection control practitioners, and lab technicians, as well as NCDPH staff.

PHEs further enhance syndromic surveillance efforts in the state by actively monitoring admissions, lab, and death reports for their hospital systems, which account for 39% of general/acute care beds in North Carolina. Although PHEs do not perform routine infection control activities, they are able, using hospital data systems, to detect and investigate unusual cases or clusters of communicable disease not only among emergency department patients, but among inpatients as well.

The findings of the study indicate that PHEs enhance communicable disease management in North Carolina by improving the completeness of communicable disease reporting. Underreporting of communicable diseases is well documented [10]. PHEs likely reduce underreporting (for the state's 11 largest hospital systems) by directly reporting cases to LHDs and NCDPH (or assisting physicians with reporting) and educating medical students, residents, and physicians on the importance of reporting. Active surveillance has been shown to enhance completeness of reporting for certain communicable diseases, including hepatitis and salmonellosis [11]. PHEs conduct surveillance on a daily basis by monitoring North Carolina's syndromic surveillance system and investigating signals and also by monitoring hospital admissions, lab, and death reports. In addition, PHEs conduct active case-finding based on alerts from LHDs and NCDPH, and during known outbreaks. Through these efforts, and by facilitating communication between the NCDPH meaningful use coordinator and appropriate hospital staff, it is likely that PHEs further reduce underreporting of communicable diseases.

Study results indicate that PHEs enhance the timeliness of public health investigations that involve cases seen at their hospital. As hospital employees serving NCDPH and LHDs as preferential partners, PHEs prioritize LHD and NCDPH requests for information needed from patient medical records. LHD respondents reported that PHEs are significantly more likely to respond "immediately" to their requests compared to staff at hospitals without PHEs. NCDPH key informants similarly highlighted PHEs ability to quickly access patient medical records and share information.

During the H1N1 pandemic, PHEs played an indispensible role in the surveillance "information loop." This loop involves healthcare facilities reporting cases to public health officials who in turn analyse case data in aggregate and develop recommendations for prevention and control. Public health officials then disseminate these recommendations back to healthcare facilities and others who are in a position to act on them, thereby completing the loop [12]. Because PHEs are based at the state's 11 largest, tertiary care/referral hospitals, where the most serious cases of H1N1 were likely to be seen, NCDPH was able to utilize these hospitals as informal sentinel surveillance sites during the pandemic. PHEs provided NCDPH with timely data on overall numbers of cases, as well as detailed information on the most severe cases. PHEs were able to quickly adapt the focus of their surveillance data to provide NCDPH with detailed case information on specific populations most severely affected by H1N1. NCDPH then used the information provided by PHEs, in conjunction with federal guidelines, to develop guidance (e.g., case definitions, testing guidelines, isolation and quarantine protocols) for healthcare providers. This guidance was disseminated back to hospitals through the PHEs, who distributed it to appropriate hospital staff and assisted in development of hospital protocols and policies related to H1N1.

The study has two main limitations. First, in gathering data from hospitals, the research team needed to limit the number of interviews to a manageable number and thus was not able to interview some hospital representatives who interact closely with PHEs, such as lab technicians and emergency department and infectious disease physicians. Therefore, the perceived value of the PHE program to hospitals presented here may be incomplete. Second, while stakeholders provided their views on the value of the PHE program, the research team could not independently confirm the accuracy of these views. However, perceptions of the value of the PHE program were consistent both within and across stakeholder groups.

## Conclusions

To effectively detect and respond to public health emergencies, hospitals and public health agencies must work together. North Carolina's hospital-based PHE program is an innovative program that links hospitals and public health agencies for enhanced syndromic surveillance, communicable disease management, and emergency preparedness and response. This description of the PHE program and its value to stakeholders, both in routine daily practice and in responding to a major public health emergency, can inform other states that may wish to establish a similar program as part of their larger public health emergency preparedness and response system.

## Competing interests

The authors declare that they have no competing interests.

## Authors' contributions

MM contributed to the design of the study; collected, analyzed, and interpreted the data; and drafted and revised the manuscript. CB and JH assisted with data collection, interpretation of data, and revising the manuscript. JAH and MD assisted with interpretation of data and revising the manuscript. PDMM contributed to the conception and design of the study and assisted with interpretation of data and revising the manuscript. All authors read and approved the final manuscript.

## Pre-publication history

The pre-publication history for this paper can be accessed here:

http://www.biomedcentral.com/1471-2458/12/141/prepub
